# Vitamin D3 administration prevents memory deficit and alteration of biochemical parameters induced by unpredictable chronic mild stress in rats

**DOI:** 10.1038/s41598-021-95850-6

**Published:** 2021-08-11

**Authors:** Hossein Bakhtiari-Dovvombaygi, Saeed Izadi, Mostafa Zare, Elham Asgari Hassanlouei, Hossein Dinpanah, S. Mohammad Ahmadi-Soleimani, Farimah Beheshti

**Affiliations:** 1 Student Research Committee, Torbat Heydariyeh University of Medical Sciences, Torbat Heydariyeh, Iran; 2 Neuroscience Research Center, Torbat Heydariyeh University of Medical Sciences, Torbat Heydariyeh, Iran; 3 Departments of Physiology, School of Paramedical Sciences, Torbat Heydariyeh University of Medical Sciences, Torbat Heydariyeh, Iran; 4 Department of Animal Biology, Faculty of Natural Sciences, University of Tabriz, Tabriz, Iran; 5 Department of Emergency Medicine, 9 Dey Educational Hospital, Torbat Heydariyeh University of Medical Sciences, Torbat Heydariyeh, Iran

**Keywords:** Biochemistry, Neuroscience

## Abstract

The present study aimed to investigate the effects of vitamin D3 (Vit D) administration on memory function, hippocampal level of amyloid-beta (Aβ), brain-derived neurotrophic factor (BDNF) and oxidative stress status in a rat model of unpredictable chronic mild stress (UCMS). Vit D was intraperitoneally administered at doses of 100, 1000, and 10,000 IU/kg. Animals were subjected to UCMS for a total period of 4 weeks. Memory function was assessed using morris water maze (MWM) and passive avoidance (PA) tests. Biochemical markers were measured to reveal the status of oxidative stress and antioxidant defense system. In addition, the levels of Aβ and BDNF were measured in hippocampal region. In the UCMS group, latency to find the platform was greater and the time spent in target quadrant (MWM test) as well as the latency to enter the dark compartment (PA test), were less than the vehicle group. Hippocampal malondialdehyde (MDA) and Aβ concentrations in the UCMS group were higher than the vehicle group. Hippocampal level of thiol and BDNF plus the activities of catalase and superoxide dismutase (SOD) were reduced in UCMS group compared to the control subjects (i.e. vehicle group). Interestingly, Vit D treatment supplementation reversed the mentioned effects of UCMS. Our findings indicated that Vit D administration improves UCMS-induced impairment of learning and memory through prevention of adverse effects on Aβ, BDNF and oxidative stress parameters.

## Introduction

In the fast-paced modern world, psychological health issues like anxiety and memory defects have become very common among the masses^1^. Exposure to stress has been shown to cause various cognitive deficits^2^. Over the past two decades, research endeavors have identified that secretion of hormones and neurotransmitters following stressful events are the major modulators learning and memory processes in humans^3^. Unpredictable chronic mild stress (UCMS) or high levels of stress hormone, which is known to inhibit hippocampal neurogenesis, are associated with intensified expression of depressive-like behaviors and cognitive impairment^4^. The structure and function of hippocampus, as a part of limbic system, undergo significant alterations following chronic mild stress^5^. UCMS may lead to a reduction in hippocampal volume due to, increased dendrites remodeling, decreased neurogenesis and loss of glial cells^6^. Previous studies have shown that a reduction in hippocampal volume can be a primary cause of impaired spatial cognition^7^. Spatial learning and memory impairments occur at the result of UCMS-induced dysregulation in level of corticosteroid and brain-derived neurotrophic factor (BDNF)^8^. Prolonged exposure to UCMS results in decreased hippocampal expression of BDNF^9^. This critical factor is known as the most abundant neurotrophin within the hippocampus^10^ and it’s main physiological functions include neuronal protection and specialization in the brain^10^. Based on the neurotrophic hypothesis, reduced expression of BDNF leads to hippocampal atrophy in response to stress^8^. On the other hand, there is a direct connection between the rate of mental illness in Alzheimer's disease (AD) patients and the number of amyloid-beta (Aβ) plaques^11^. Aggregation of Aβ1-42 in senile plaques leads to neuronal apoptosis, oxidative stress and memory dysfunction^12^. In addition, it is now well-etablished that chronic stress can speed up the development and progression of AD by increasing the synthesis of amyloid precursor protein (APP)^13^.

Vitamin D3 (Vit D), is able to cross the blood–brain barrier (BBB) and its receptors are widely distributed in the central nervous system. It has been shown that Vit D supplementation improves cognitive performance, more significantly attention and memory^14^. Chronic deficiency of Vit D may accelerate the process of neuronal degeneration and cognition defects in AD pateints^15^. Consistently, Vit D reduces oxidative stress and, as shown in the literature, prevents neuronal death by activating macrophages which in turn promote the elimination of Aβ plaques^16^. In addition, it has been shown that Vit D treatment increases the level of BDNF expression in aged rats^17^ as well as in the hippocampus of diabetic animals^18^. Regarding the mentioned neuroprotective effects, the present study was conducted to evaluate the effects of Vit D supplimentation on BDNF expression, Aβ formation, oxidative stress balance and the UCMS-induced impairment of learning and memory in rats.

## Results

### Vit D reduced UCMS-induced enhancement of serum corticosterone level

**Figure 1:**
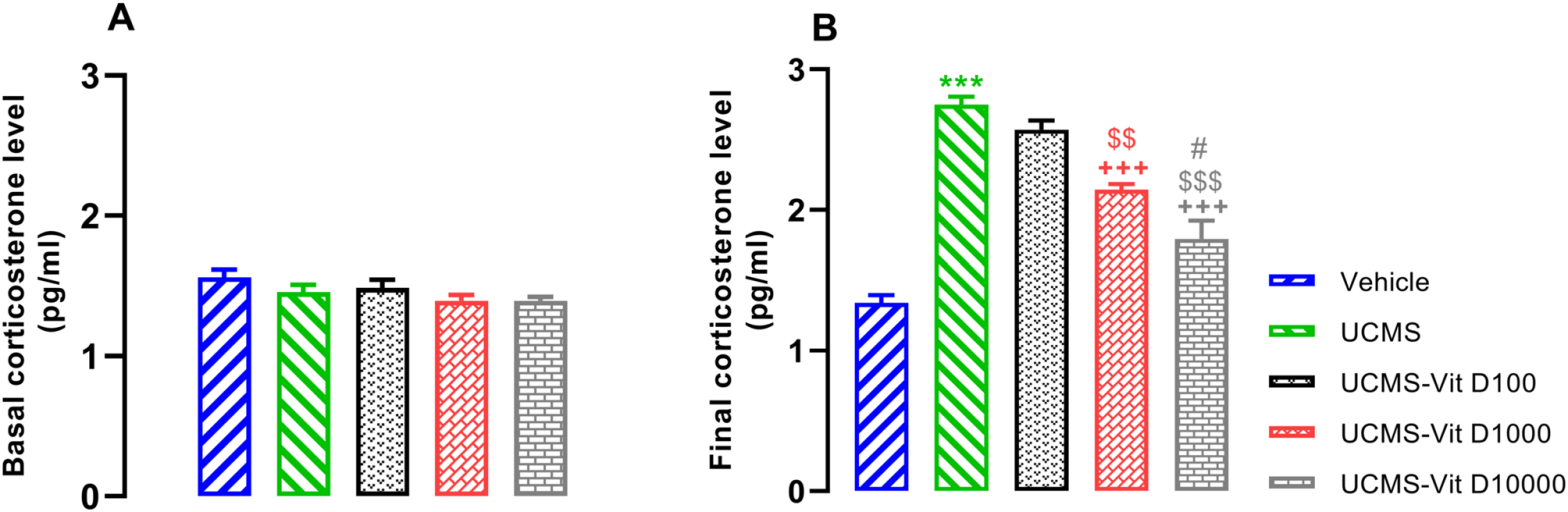
Serum corticosteone level in experimental groups. (**A**) Basal (pre-stress) and (**B**) final (post-stress) level of corticosteone. Data are presented as mean ± SEM, n = 10. ***P < 0.001 versus Vehicle group, +  +  + *P* < 0.01 versus UCMS group, ^$$^*P* < 0.01 and ^$$$^*P* < 0.001 versus UCMS-Vit D100 group and ^#^*P* < 0.05 0 versus UCMS-Vit D1000 group.

The obtained results showed that four weeks application of UCMS results in increased serum corticosterone level (shown as the final level) in rats compared to the vehicle group (Fig. 1B, *P* < 0.001). Furthermore, this effect was attenuated in animals that received Vit D (1000 and 10,000 IU/kg, *P* < 0.001). In this respect, it should be noted that the effect of Vit D displayed a dose-dependent profile, i.e., attenuation of corticosterone level was more significant following administration of higher doses (Vit D 100 vs. 1000, *P* < 0.01, Vit D 100 vs. 10,000, *P* < 0.001, Vit D 1000 vs. 10,000, *P* < 0.05). Interestingly, corticosterone level prior to the application of UCMS protocol (shown as the basal level) was not significantly altered among different groups (Fig. 1A).

### Vit D improved UCMS-induced impairment of spatial learning and memory

**Figure 2:**
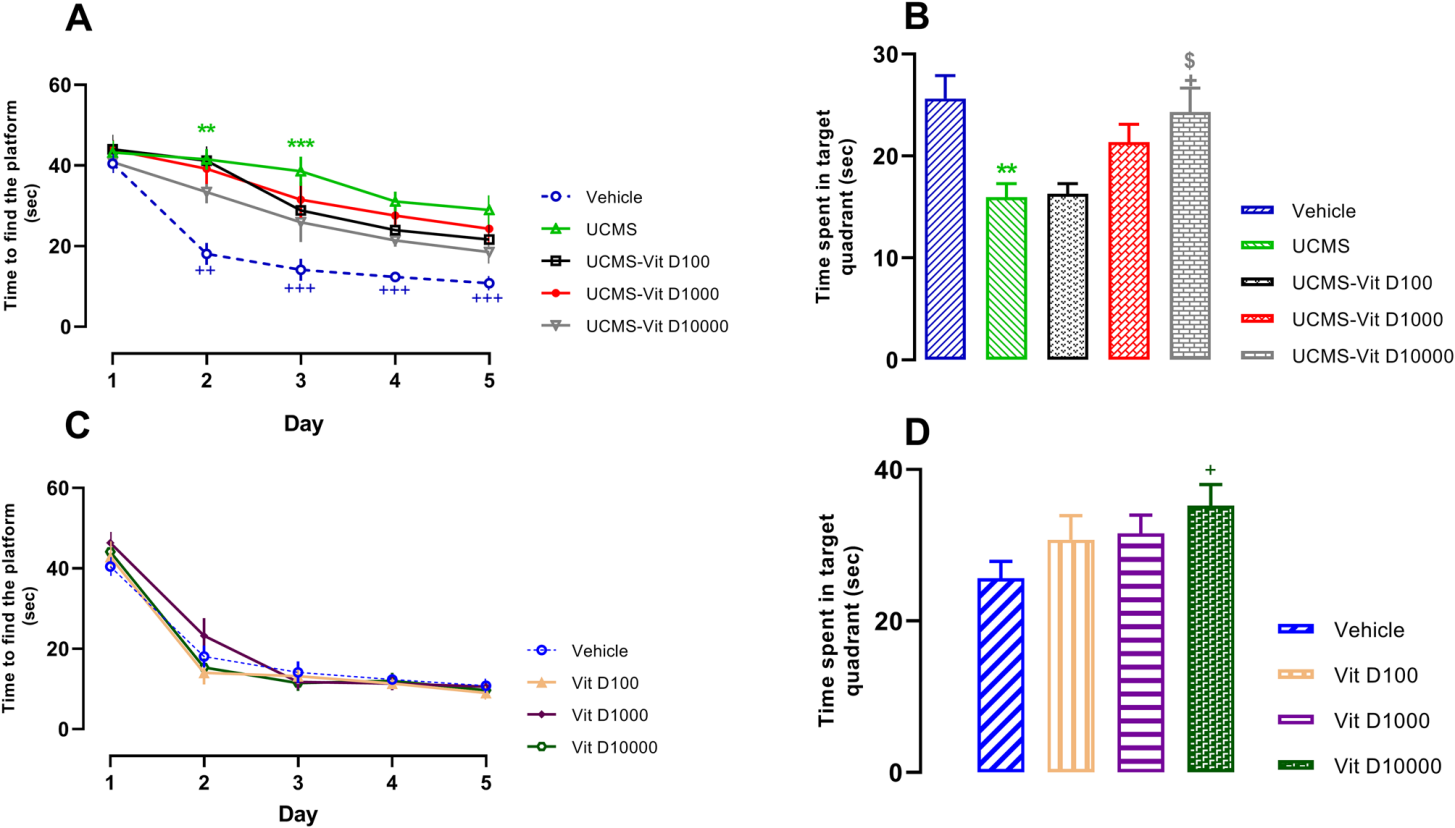
Comparison of MWM test results in different groups. (**A**) time to find the platform in UCMS groups. (**B**) time spent in target quadrant in UCMS groups. (**C**) time to find the platform in Vit D groups and (**D**) time spent in target quadrant in Vit D groups. Data are presented as mean ± SEM, n = 10. For A and B sections: +  + *P* < 0.01 and +  +  + *P* < 0.001 versus day 1 in Vehicle group ** *P* < 0.01 and ***P < 0.001 versus Vehicle group, + *P* < 0.05 versus UCMS group and ^$^*P* < 0.05 versus UCMS-Vit D100 group. For D section: + *P* < 0.05 versus vehicle group.

As indicated in Fig. 2A, when assessed in MWM, time to find the platform was progressively decreased during 5 days, representing gradual development of learning in rats. This effect was significant on day 2 and further potentiated on days 3, 4 and 5 (*P* < 0.01, *P* < 0.001, respectively). In addition, four weeks application of UCMS prevented the reduction of latency (i.e. attenuated learning) which was statistically significant on days 2 and 3 (*P* < 0.01, *P* < 0.001, respectively). However, Vit D treatment, regardless of its dosage, did not affect UCMS-induced learning impairment. As for the results of probe day, we observed that in UCMS group, the time spent in the target quadrant was significantly reduced compared to the vehicle group, indicating the induction of memory impairment (Fig. 2B, *P* < 0.01). Interestingly, Vit D treatment at the dose of 10,000 IU/kg increased the duration of animal swiming in the target zone, compared to the UCMS group and lower doses of Vit D (*P* < 0.05). Finally, following the application of Vit D in normal (without UCMS) rats, the decremental trend of time to find the platform (as an index of learning) was not altered, compared to vehicle group (Fig. 2C). As for the memory assessment, only the highest dose of Vit D (10,000 IU/kg) could increase the “time spent in targrt quadrant”, indicating improvement of memory function, compared to the vehicle group (Fig. 2D, *P* < 0.05).

### Vit D improved UCMS-induced impairment of passive avoidance memory

**Figure 3:**
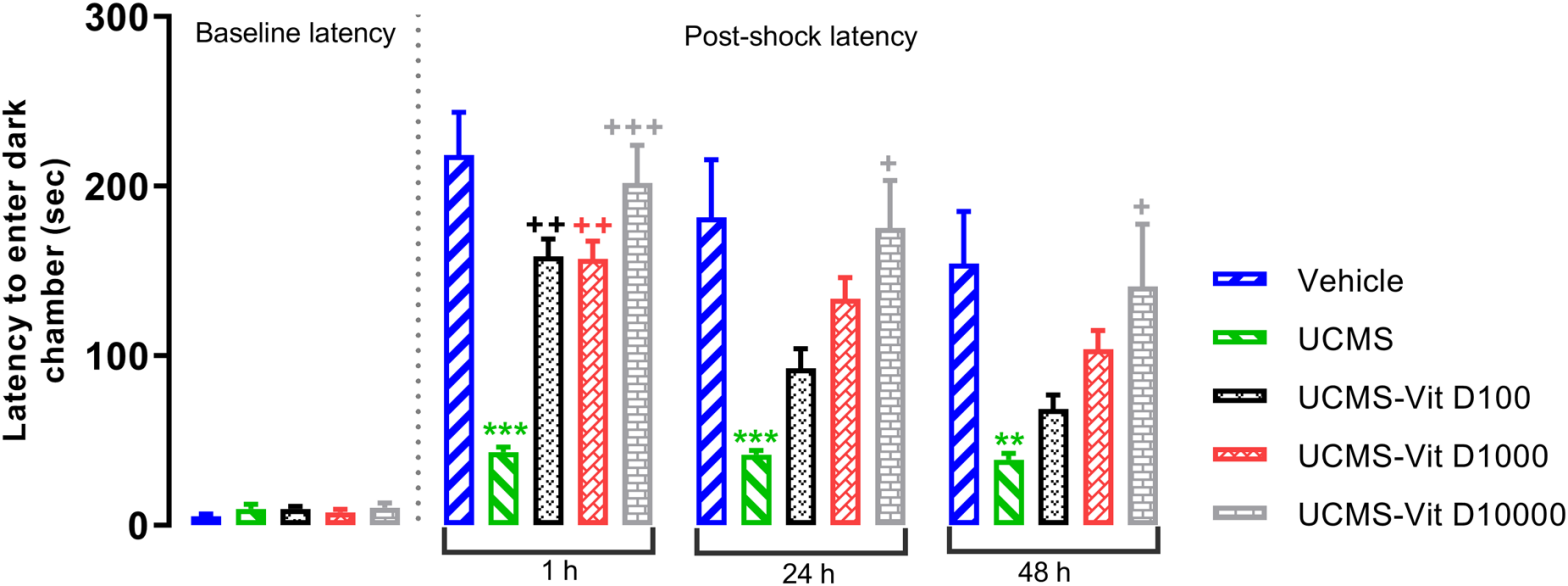
Comparison of latencies to enter dark chamber in UCMS groups. Data are presented as mean ± SEM, n = 10. **P < 0.01 and ***P < 0.001 versus Vehicle group, + *P* < 0.05, +  + *P* < 0.01 and +  +  + *P* < 0.001 versus UCMS group.

**Figure 4:**
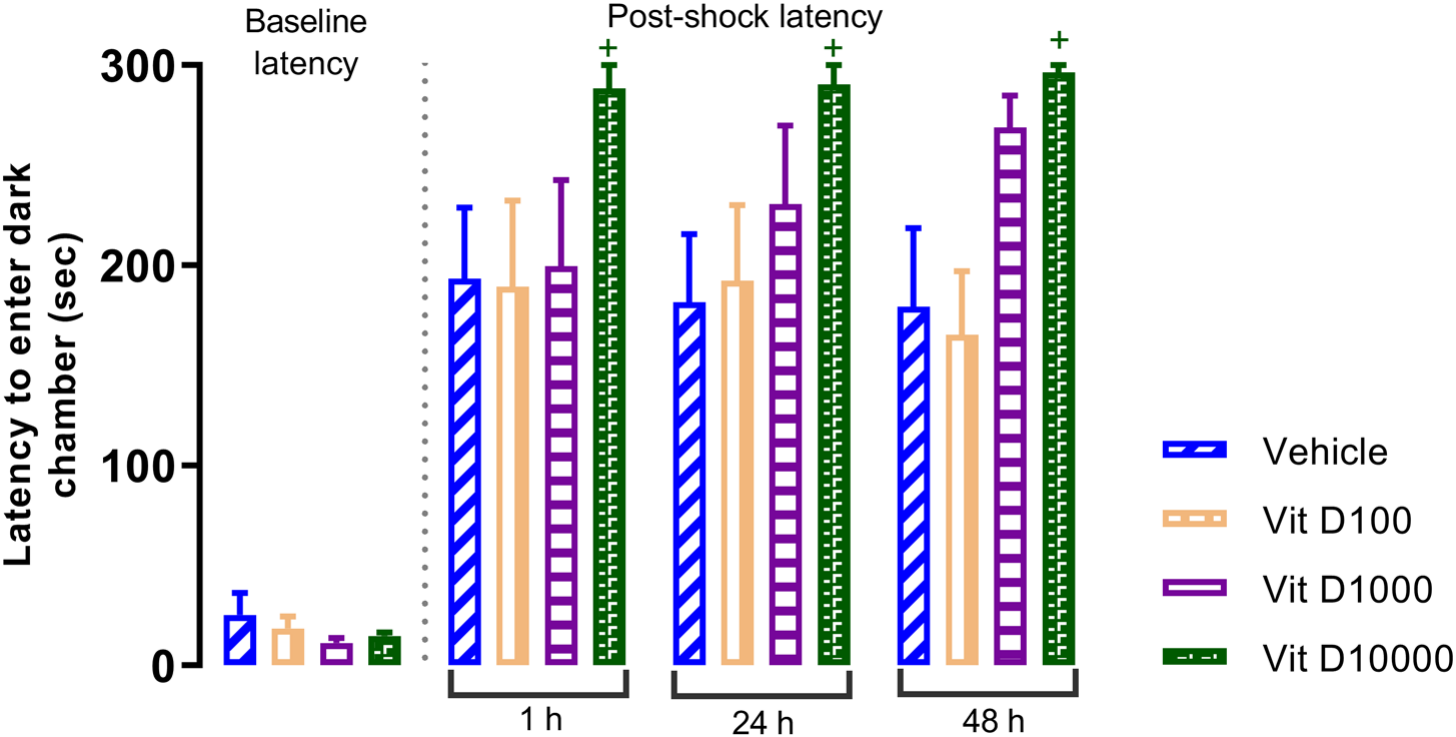
Comparison of latencies to enter dark chamber in Vit D groups. Data are presented as mean ± SEM, n = 10. + *P* < 0.05 versus Vehicle group.

Results of PA test revealed that four weeks application of UCMS significantly decreases the “latency to enter the dark chamber” 1, 24 and 48h after electrical shock (Fig. 3). *P*<0.001, *P*<0.001 and *P*<0.01, respectively). One hour after the shock, all Vit D doses significantly increased the latency (*P*<0.01, *P*<0.01, *P*<0.001 for 100, 1000 and 10000 IU/kg, respectively). However, 24 and 48h later, the mentioned effect was only observed for the highest dose, i.e., 10000 IU/kg (*P*<0.05). We should mention that, the experimental groups were not different in response atency prior to reciving the shock. Highest dose of Vit D (10000 IU/kg) in naïve rats caused enhancement of “latentcy to enter the dark chamber”, indicating improvement of passive avoidance memory (Fig. 4, *P*<0.05).

### Vit D improved UCMS-induced disturbance of oxidant/antioxidant balance in hippocampal tissues

**Figure 5:**
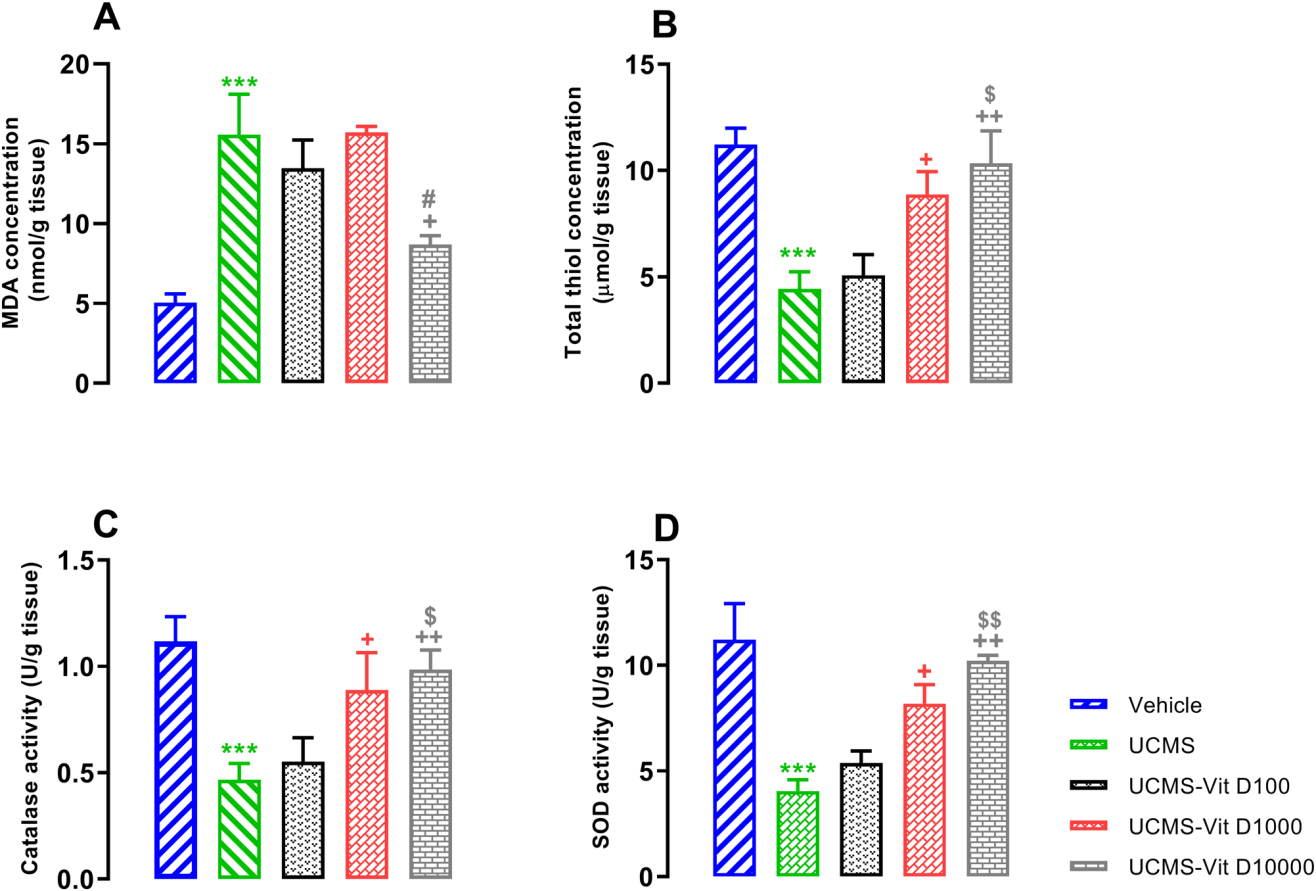
Hippocampal concentrations of MDA (**A**), thiol content (**B**) and activities of catalase (**C**) and SOD (**D**) in UCMS groups. Data are presented as mean ± SEM, n = 10. ***P < 0.001 versus Vehicle group, + *P* < 0.05 and +  + *P* < 0.01 versus UCMS group, ^$^ *P* < 0.05 and ^$$^ *P* < 0.01 versus UCMS-Vit D100 group and # *P* < 0.05 0 versus UCMS-Vit D1000 group.

**Figure 6:**
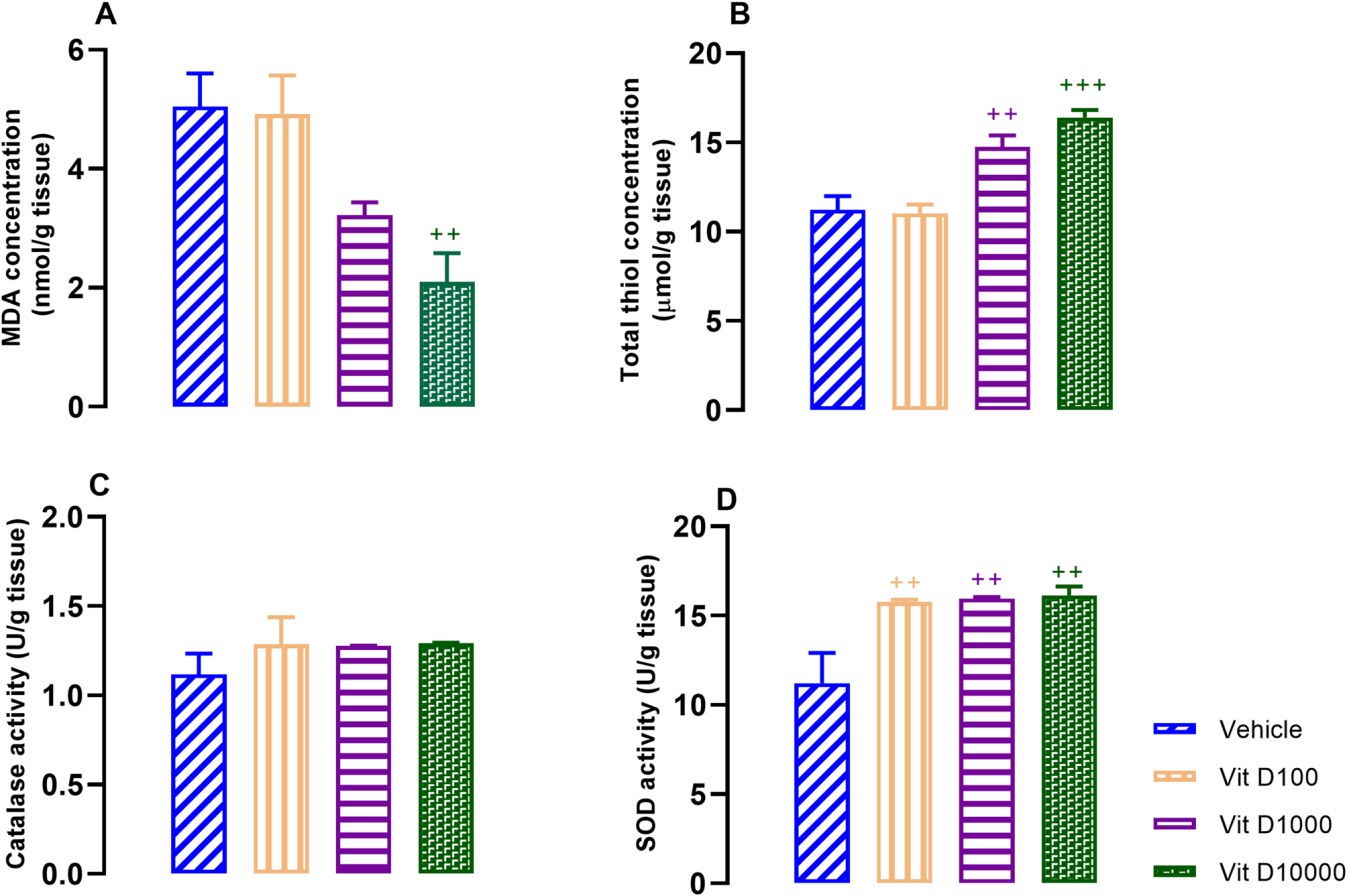
Hippocampal concentrations of MDA (**A**), thiol content (**B**) and activities of catalase (**C**) and SOD (**D**) in Vit D groups. Data are presented as mean ± SEM, n = 10. +  + *P* < 0.01 and +  +  + *P* < 0.001 versus vehicle group.

UCMS induction elevated MDA concentration and reduced thiol content as well as catalase and SOD activity (Fig. 5, *P*<0.001). Vit D, at the dose of 10000 IU/kg reduced UCMS-induced elevation of MDA concentration, compared to the UCMS group (Fig. 5A, *P*<0.005). Moreover, doses of 1000 and 10000 increased the total thiol content and catalase/SOD activity, compared to the UCMS group (Fig. 5B–D, *P*<0.05 and *P*<0.01, respectively). It should be noted that the highest dose of Vit D (10000 IU/kg) decreased the MDA level compared to vehicle group (Fig. 6A, *P*<0.01). On the other hand, doses of 1000 and 10000 of Vit D increased the total thiol content (Fig. 6B, *P*<0.01 and *P*<0.001, respectively). In case of catalase activity, no difference was observed among the experimental groups (Fig. 6C). Finally, all doses of Vit D increased SOD activity in naïve rats, compard to vehicle group (Fig. 6D, *P*<0.01).

### Vit D reversed UCMS-induced changes of BDNF and Aβ concentration in hippocampal tissues

**Figure 7:**
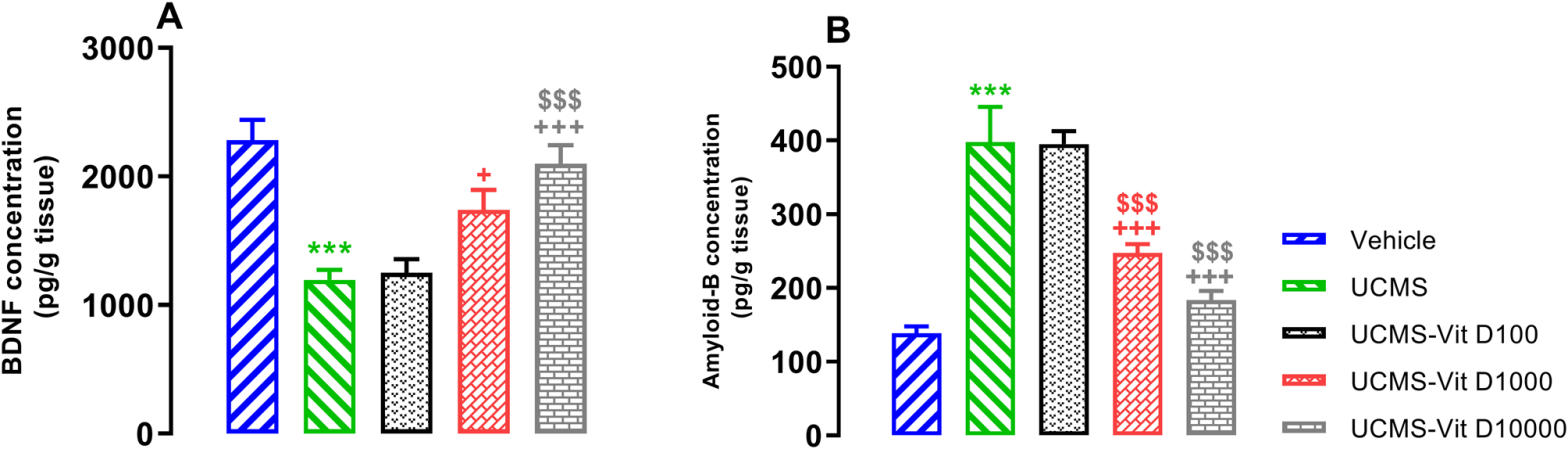
Hippocampal concentrations of Aβ (**A**) and BDNF (**B**). Data are presented as mean ± SEM, n = 10. ***P < 0.001 versus Vehicle group, + *P* < 0.05 and +  +  + *P* < 0.001 versus UCMS group, ^$$$^ *P* < 0.001 versus UCMS-Vit D100 group.

Application of UCMS reduced hippocampal BDNF concentration (*P*<0.001) and this effect was prevented in animals that had recived Vit D 1000 and 10000 IU/kg (Fig. 7A, *P*<0.05 and *P*<0.001). Again, UCMS caused elevation of Aβ level in hippocampal tissues (Fig. 7B, *P*<0.001) and this effect was prevented by the two higher doses of Vit D (i.e. 1000 and 10000 IU/kg, both *P*<0.00).

### Vit D supplementation reversed UCMS-induced reduction of serum 25 (OH) Vit D3 level

**Figure 8:**
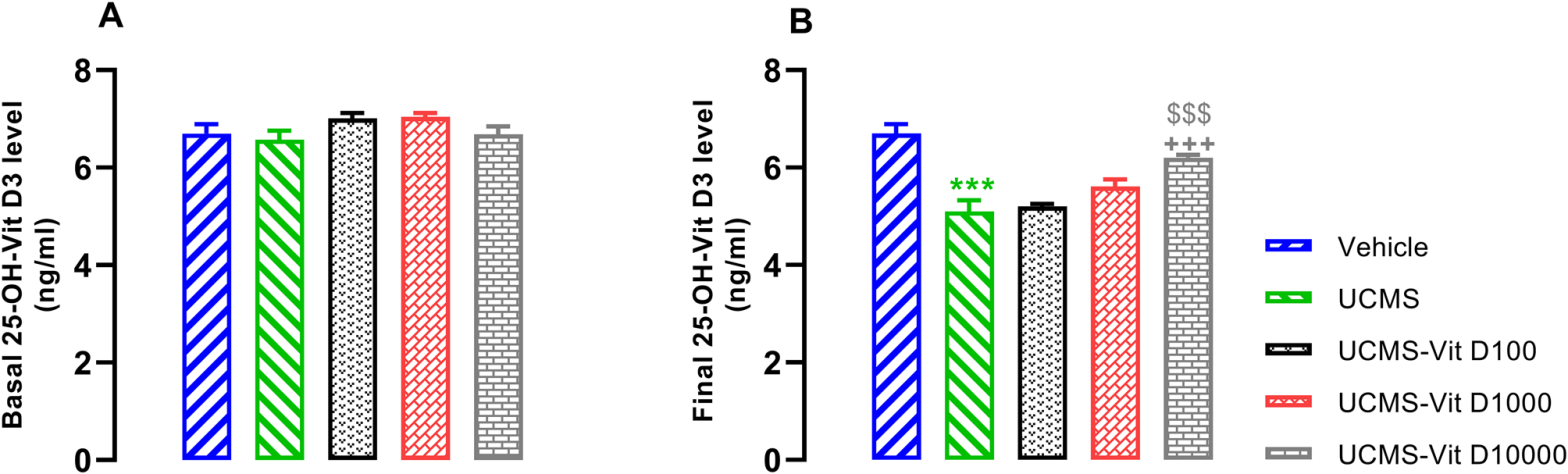
Basal (**A**) and final (**B**) levels of Vit D in UCMS groups. Data are presented as mean ± SEM, n = 10. ***P < 0.001 versus Vehicle group, +  +  + *P* < 0.001 versus UCMS group, ^$$$^*P* < 0.001 versus UCMS-Vit D100 group.

Basal level of 25 (OH) Vit D3 was not significantly different among the experimental groups (Fig. 8A), however, the final (post-UCMS) concentration was reduced compared to the vehicle group (Fig. 8B, *P* < 0.001). Vit D supplementation, dose dependently, reversed UCMS-induced suppression of serum 25 (OH) Vit D3 level ((Fig. 8B, *P* < 0.001).

### Correlation between Vit D doses and oxidant/antioxidant parameters in hippocampal tissues

**Figure 9:**
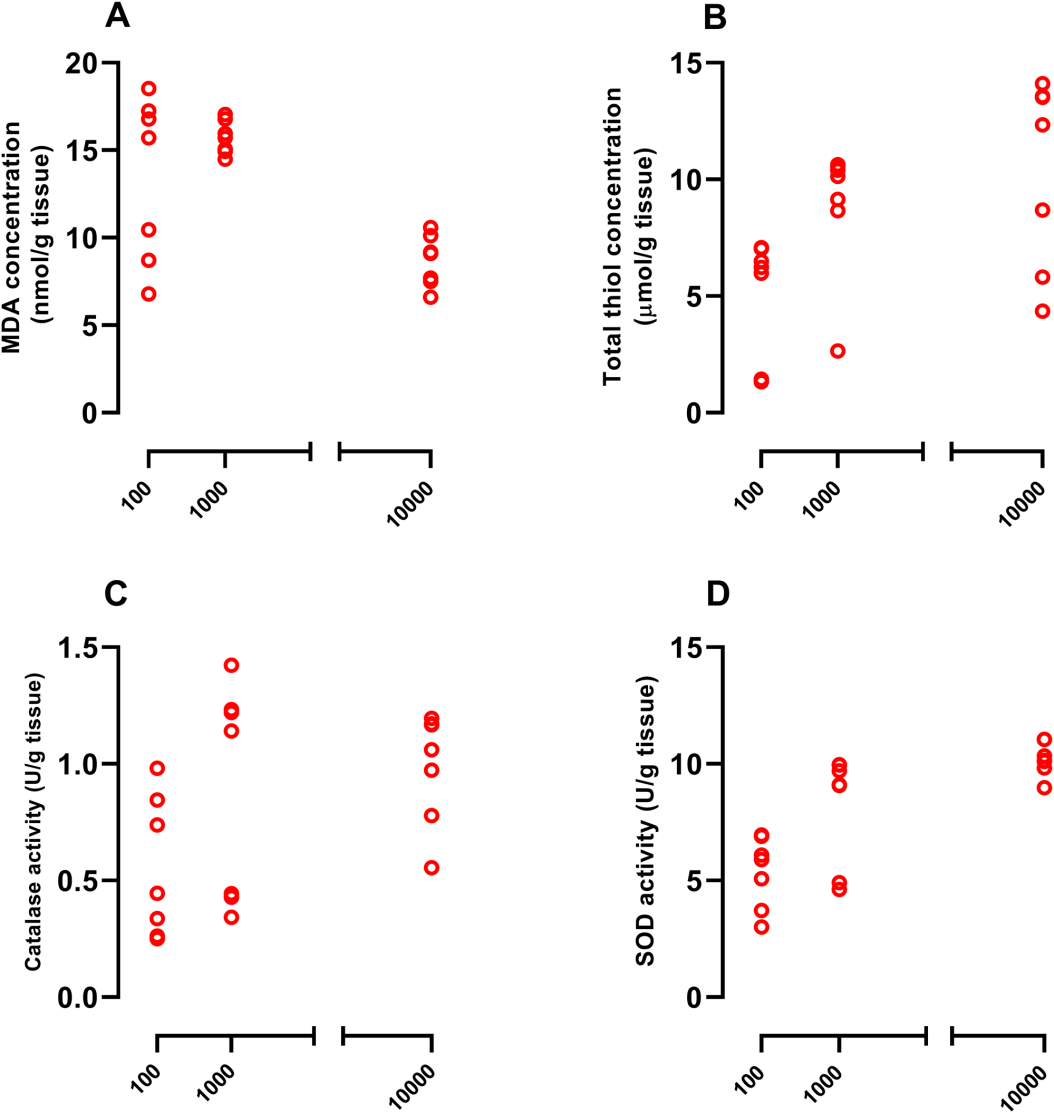
Correlation of Vit D doses with MDA (**A**), total thiol (**B**), catalase (**C**) and SOD (**D**). As indicated, there is a positive correlations between doses of Vit D and total thiol content and SOD activity (Pearson r = 0.4596, *P* < 0.05 and Pearson r = 0.6767, *P* < 0.001, respectively), however, this was found to be negative in case of MDA levels (Pearson r =  − 0.6799, *P* < 0.001). No significant correlation was found for catalase activity (Pearson r = 0.3658 and *P* = 0.1029).

Results showed that there is a significant correlation between 3 doses of Vit D-MDA and Vit D-total thiol concentration in the UCMS groups (Pearson r =  − 0.6799, *P* < 0.001, Fig. 9A and Pearson r = 0.4596, *P* < 0.05, Fig. 9B, respectively). Furthermore it was observed that the correlation between all doses of Vit D with SOD activity is significant (Pearson r = 0.6767, *P* < 0.001, Fig. 9D). Additonally, the results showed that there is no significant correlaton between catalase activity and 3 doses of Vit D (Fig. 9C).

### Correlation between Vit D doses and BDNF/Aβ concentration in hippocampal tissues

**Figure 10:**
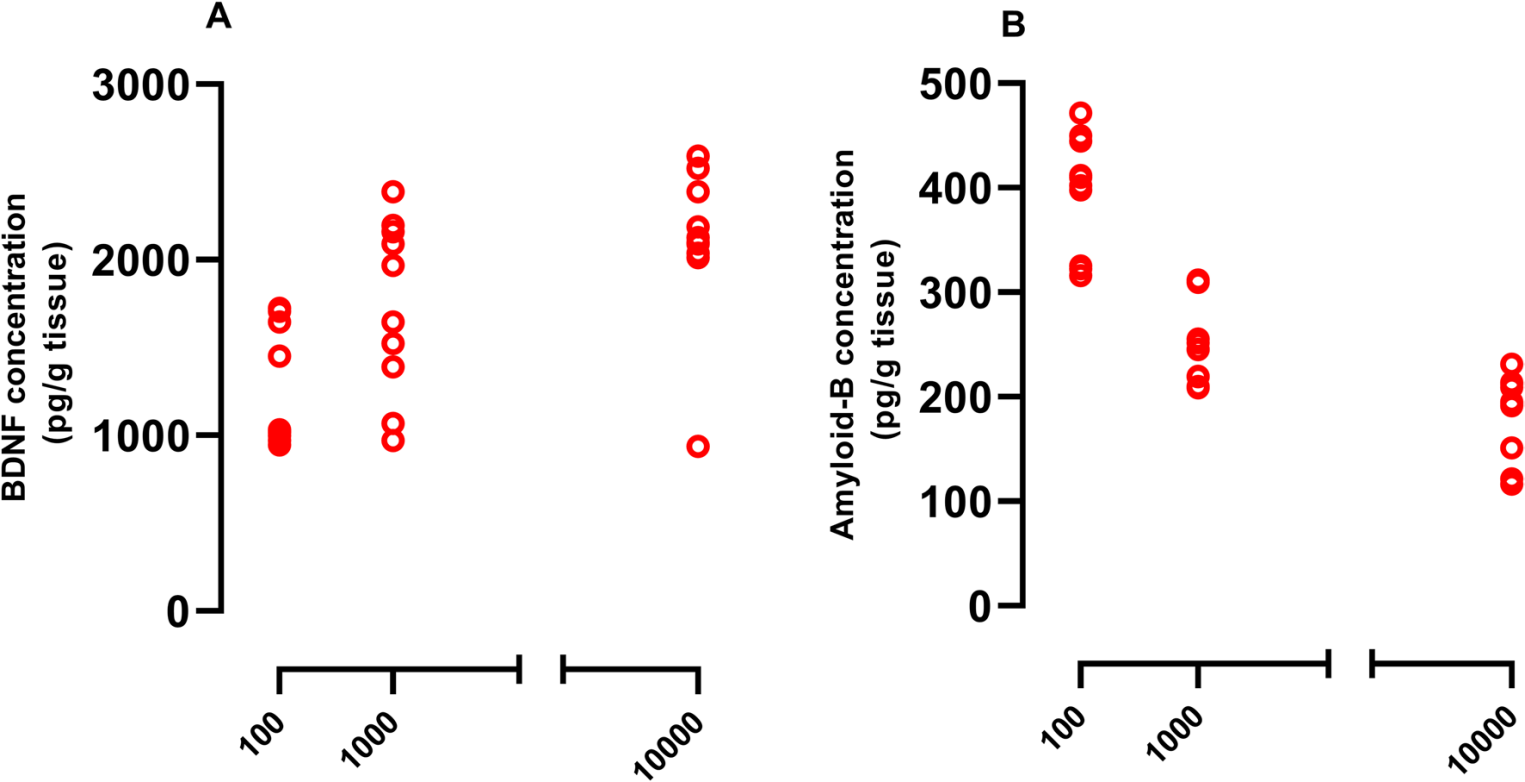
Correlation of Vit D doses with BDNF (**A**) and Aβ (**B**). As indicated, there is a positive correlations between doses of Vit D and BDNF (Pearson r = 0.5562, *P* < 0.01), however, this was found to be negative in case of Aβ levels (Pearson r =  − 0.7080, *P* < 0.001).

Significant correlation was found between all doses of Vit D (100, 1000 and 10,000) with BDNF and Aβ concentrations (Pearson r = 0.5562, *P* < 0.01, Fig. 10A and Pearson r =  − 0.7080, *P* < 0.001., Fig. 10B, respectively).

## Discussion

During the last decade, several studies have addressed the adverse effects of chronic stress (as UCMS model) on learning and memory^19^, however, the underlying mechanism/s have not been well investigated. On the other hand, the improving effects of Vit D on learning and memory is well established. These evidence motivated us to investigate whether Vit D administration could prevent the detrimental effects of UCMS at cellular and behavioral levels.

In the present study, our findings demonstrated that treatment of rats with Vit D significantly prevents the UCMS-induced impairment of learning and memory. Moreover, Vit D administration in rats, leads to a remarkable reduction of Aβ level and a dramatic increase in expression of BDNF within the hippocampal tissues. Long-term exposure to UCMS has been shown to cause memory and learning impairments^20,21^. Inerestingly, we found that this adverse effect is associated with an increase in corticosterone level. It is well known that corticosterone secretion promotes the oxidative stress, which in turn leads to memory impairment^22,23^. In this study, UCMS caused brain oxidative damage by reduction of the total thiol concentration, suppression of CAT/SOD activity and enhancement of the MDA level. Consistentely, Abelaira et al. (2013) have demonstrated a significant reduction of SOD/CAT and increased level of MDA in the prefrontal cortex of stressed rats^24^. Furthermore, our results indicated that UCMS decreases the BDNF level in hippocampal tissues of rats. BDNF is well known among the researchers to have a crucial role in synaptic plasticity which is as an essential mechanism for development of learning and memory^25,26^. In previous stuides, UCMS has been shown to reduce the BDNF level and cause memory deficits^27^. In addition, a negative correlation has been reported between oxidative stress and BDNF level^28^. Therefore, any defect in this factor might be somehow associated with the learning and memory impairment in the UCMS model.

In this study, we have also assessed the hippocampal level of Aβ in animals undergone the UCMS protocol. In this regard, there is evidence indicating that stress can increase Aβ production^29^. As aforementioned, accumulation of Aβ in brain results in neuronal death, oxidative stress and memory dysfunction. In our experiments, UCMS increased the Aβ level in hippocampus of animals displaying impaired learning and memory. Thus, Aβ might be a key mediator through which chronic stress results in cognitive deficits.

Numerous studies have suggested the involvement of Vit D in promotion of cognitive functions and neuroprotection^30–32^. Epidemiological studies have also shown a direct correlation between Vit D levels in the serum and improvent of memory test performance^30^. Moreover, a growing body of evidence have revealed that low Vit D levels are directly associated with increased severity of AD and dementia incidents^33–35^. In this study, it is demonstrated that Vit D prevents the learning/memory impairment induced by UCMS in rats. In theVit D-treated groups, animals exhibited shorter escape latencies than the UCMS group, when examined by the MWM test. Furthermore, the Vit D-treated rats showed better behavioral performance in the probe trials (24 h later) which indicates improvement of memory retrieval. A similar effect was observed in the passive avoidance test. Vit D prevented UCMS-induced cognitive deficits in retention trials; 1, 24 and 48 h later which indicates the improvement of memory function. It has been shown that Vit D deficiency during the early developmental steps could alter the fetal glucocorticoid exposure^36^. Our data also showed that Vit D treatment partialy nullified the effects of UCMS on serum corticosterone levels. The positive effects of Vit D on learning and memory might be attributable to this effect.

**Figure 11:**
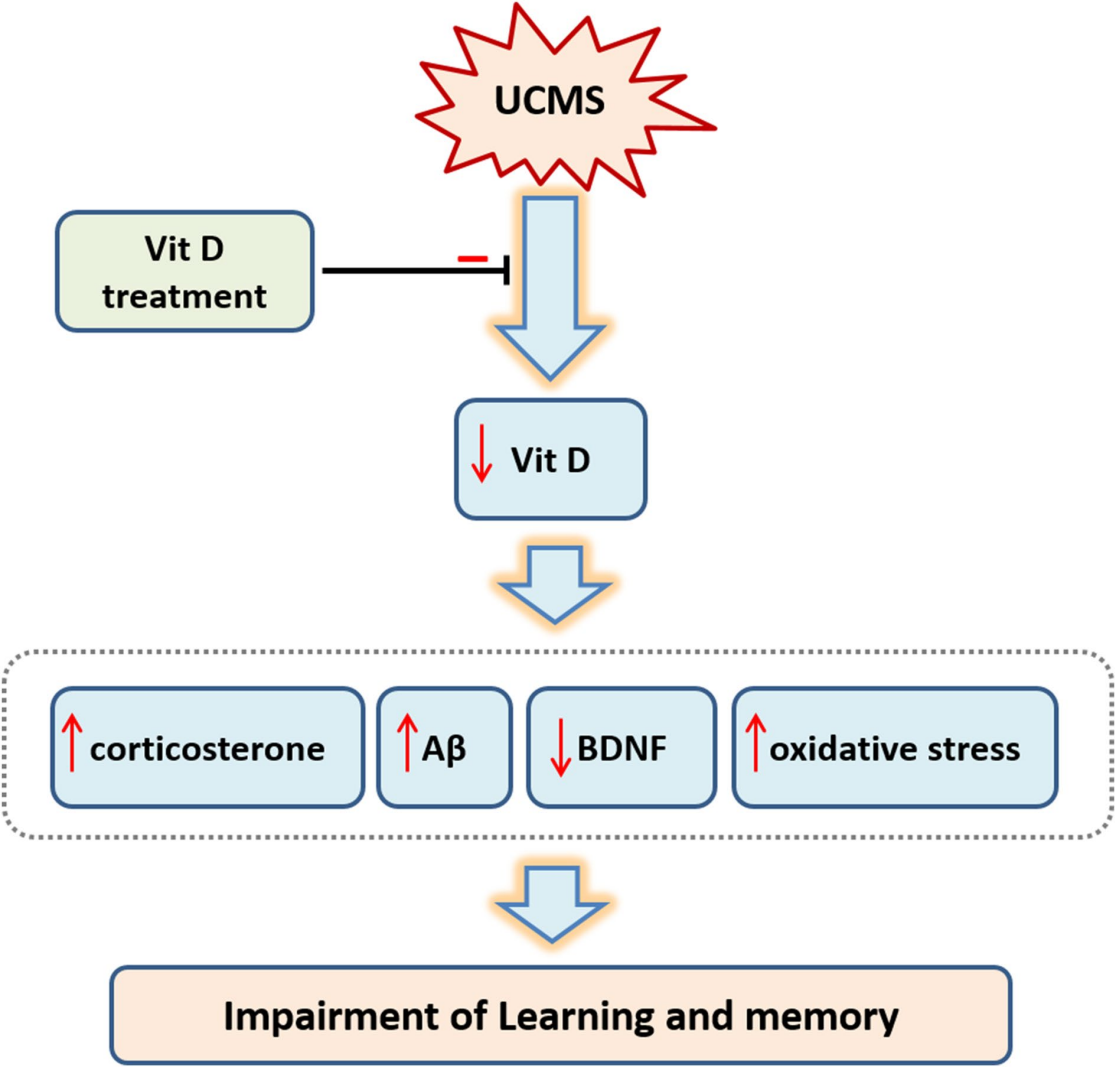
Hypothetical model indicating several machanisms through wich UCMS causes impairment of learining and memory and the protective effect of Vit D treatment. As indicated, UCMS causes Vit D deficiency which in turn leads to the following changes in: increased level of corticosterone, Aβ and oxidative stress markers, as well as reduced level of BDNF. In addition, Vit D treatment can prevent the mentioned UCMS-induced adverse effects.

Previous studies have shown that Vit D induces protection against oxidative stress through upregulation of antioxidant proteins^37^. In this study, administration of Vit D in normal rats significantly decreased the MDA level which represents the suppression of oxidative stress. It also increased the total thiol content and SOD activity, which indicates promotion of antioxidant defense mechanisms in the hippocampus. Thses findings might explain the basic mechanisms underlying our behavioral observations, i.e. increased latency to enter the dark chamber and increased time spent in tagrt quadrant which represent the improved avoidance and spatial memory, respectively (Figs. 2D, 4). Consistently, what we report here as the preventive effect of Vit D on UCMS-induced cognitive impairment might be associated with attenuation of oxidative stress damage in brain (Fig. 11).

In Vit D-treated groups, increased level of BDNF was associated with a significant decrease in Aβ concentration in hippocampus. A similar study has shown that Vit D can increase the BDNF level in UCMS depression model^38^. Given these findings; it could be proposed that Vit D treatment could improve learning/memory through potentiation of BDNF synthesis and inhibition of Aβ formation (Fig. 11). Although, Vit D's mechanism of action for re-establishing the hippocampal BDNF level is still poorly understood, we found a direct association between Vit D treatment and BDNF levels. Morover, our results raises the possibility that BDNF may alter the metabolism of free radicals such that increased cellular content of SOD attenuates the accumulation of free radicals and protects neurons from free radical attack^39^. It is known that Aβ could induce alzheimer-like signs in animal models. In this regard, there are evidence indicating that injection of Aβ causes reduction of BDNF level^40^. In vitro experiments have shown that this effect is mediated via downregulation of BDNF synthesis pathway^41^. This raises the possibility that UCMS-induced elevation of Aβ could suppress BDNF synthesis in hippocampal tissues. In addition, Vit D has been shown to inhibit the brain Aβ levels^42^, which in turn could increase the BDNF synthesis and improve the behavioral parameters of learning and memory, as we observed in our study.

Pearson's correlation test was done for biochemical parameters in UCMS groups. Significant correlation was found between Vit D doses and MDA, thiol, SOD, Aβ and BDNF. This indicates that increase in Vit D dose progressively reduces the detrimental effects of UCMS on hippocampal tissues.

Finally, we need to discuss the effect of stress on serum Vit D level. In this respect, we observed that the serum level of Vit D is almost similar prior to UCMS induction in all groups (Fig. 8A). However, following application of UCMS, level of Vit D was significantly reduced (Fig. 8B). This adverse effect was reversed by the highest dose of Vit D (10,000 IU/Kg). Consistent to this finding, previous studies have shown that Vit D deficiency is associated with stress^43^. Thus, it is possible that UCMS-induced impairment of learning and memory (as we observed in our expeiments) might be, at least in part, the result of Vit D reduction.

## Conclusion

In conclusion, our results showed that Vit D supplementation could prevent the UCMS-induced learning and memory impairment in rats. Cellular mechanisms are thought to be enhancement of BDNF concentration, reduction of Aβ level and attenuation of oxidative stress damage.

## Materials and methods

### Animals, drugs and experimental groups

Fifty adult male Wistar rats (weighing 250 ± 10) were obtained from the animal house located in Torbat Heydariyeh University of Medical Sciences. Animals were kept in plexiglass cages and maintained under constant room temperature (22 ± 2° C) with a 12-h light/dark cycle and ad libitum access to food and water (Lights on at 6:00 AM). All experimental protocols were approved by the Ethics Committee at Torbat Heydariyeh University of Medical Sciences (Ethics code for this study: IR.THUMS.REC.1399.019). Attempt was made to conduct all protocols in accordance with the ARRIVE guidelines. All methods were performed in accordance with the relevant guidelines and regulations.

Vit D (Iran Hormone Pharmaceutical Co, Tehran, Iran, 1 mL vial) was first dissolved in dimethyl sulfoxide (DMSO) 3% and then diluted with physiological saline (NaCl 0.9%) to the final concentrations of 100, 1000 and 10,000 IU/Kg. Each day, the required amount of Vit D solution was freshly prepared prior to the experiments.

**Figure 12:**
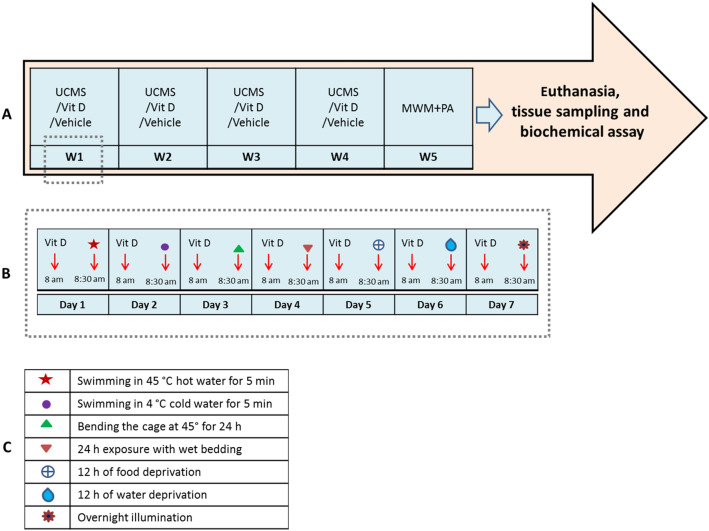
Schematic timeline indicating the sequence of experimental protocol. (**A**) Application of UCMS/Vit D administration followed by behavioral and biochemical assessments during 4 weeks, (**B**) Timing and pattern of Vit D/UCMS application during one week and (**C**) various types of stressors used in this study.

The animals were randomly assigned to control (vehicle) and experimental groups as follows: (1) Vehicle: Rats received Vit D solvent, as described earlier (NaCl 0.9% plus DMSO 3%) intraperitoneally (IP). (2) UCMS: Rats received Vit D solvent and underwent UCMS procedure as will be described later. (3) UCMS-Vit D 100: During the UCMS procedure, animals received daily IP injection of Vit D 100 IU/kg. (4) UCMS-VD 1000: During the UCMS procedure, animals received daily IP injection of 1000 IU/kg Vit D. (5) UCMS-Vit D 10,000: During the UCMS procedure, animals received daily IP injection of 10,000 IU/kg Vit D (Fig. 12)^44–46^. (6–8) Vit D100/1000/10,000: animals received daily IP injection of Vit D 100/1000 or 10,000 IU/kg during 4 weeks, respectively.

### UCMS procedure

UCMS protocol is widely being used as a well-established method for induction of long-term stress in animal models and it is thought to resemble the unavoidable stressors of daily life in humans. UCMS includes a series of unpredictable stressors^47^, some which were applied during 4 weeks in our study as follows:

1. Bending the cage at 45° for 24 h
–24 h contact with wet bedding
3. Swimming in 4 °C water for 5 min
4. Swimming in 45 °C water for 5 min
5. Overnight illumination.
–12 h of food deprivation
–12 h of water deprivation

It should be noted that the application of mentioned stressors were randomly distributed throughout each week (Fig. 12B). It was also made sure that the rats do not receive the same stressors in two consecutive days. This was done to avoid memory formation in relation with the sequences of various stressors. In other words, this method causes the rat to face an unexpcted stressor each day. The rats were stress-free and had equal access to food and water in the vehicle group^47^. Following the end of UCMS protocol, behavioral experiments, including morris water maze (MWM) and passive avoidance (PA) tests, were conducted (Fig. 12A). Finally, the rats were anesthetized by IP injection of urethane and sacrificed on the last day. Hippocampal tissues were rapidly removed for biochemical assessments.

### Behavioral assessment

#### Morris water maze (MWM) test

The MWM test was performed to assess the animal functions associated with spatial learning and memory. These included “space navigation” and “spatial probe” tests. The apparatus was a circular pool of 150 cm in diameter and 60 cm high and was filled with warm (22 ± 1 °C) water. The escape platform was 9 cm in diameter and 1.0 cm below the water surface. The space navigation test was done during 5 consecutive days, with four trials per day. If the rat failed to find the platform within 60 s, the latency time was recorded as 60 s, and the rat was gently guided to the platform by the experimenter. After the space navigation test, the spatial probe test was performed and the rats were allowed to swim for 60 s in 4 trial. The time spent in the target quadrant was recorded. All tests were performed by the same experimenter who was blind to the study design and experimental groups^48^.

#### Passive avoidance (PA) test

The device was divided by a guillotine door into light and dark zones. The animals were first placed into the apparatus to move freely between the two spaces for 5 min when the guillotine door was opened. An electric shock (2 mA for 2 s) was applied to the rat’s paw in an acquisition experiment when they entered the darkroom. After 1, 24 and 48 h, animals were placed in the light chamber and the latency to enter the dark chamber was recorded^48^.

### Biochemical assay

The animals were anesthetized by the urethane and sacrificed after the last day of the behavioral tests. Blood was collected by cardiac puncture for subsequent analyses of corticosterone serum levels. The hippocampal tissues were then removed. BDNF, Aβ, total thiol, malondialdehyde (MDA), concentration and superoxide dismutase (SOD), and catalase activities were detected hippocampal tissues.

### *Measurement of corticosterone and* 25 (OH) Vit D3 *levels*

The specific ELISA kit for serum corticosterone level (MyBiosource Co, San Diego, CA, USA) was used and the instruction provided by the manufacturer was followed. In addition, levels of 25 (OH) Vit D3 were measured using HPLC method^49^.

#### Determination of MDA and total thiol concentration

As lipid peroxidation indicator, MDA concentration in hippocampal and cortical tissues was calculated according to a previously mentioned protocol^48^. MDA reacts with thiobarbituric acid (TBA) to form a red complex. To measure the total thiol concentration, we read the absorbance of yellow complex that forms when reaction occurs between DTNB (2,2'-dinitro-5,5'-dithiol benzoic acid) and thiol groups in tissues^48^.

#### Enzymatic assessment

The method used for measurement of SOD and CAT activity has previously been explained^48^. The SOD operation was measured using the Madesh and Balasubramanian process^50^. The enzyme activity was measured at 570 nm according to a colorimetric technique. One unit of SOD is equal to the amount of enzyme that should be inhibited by 50% of the MTT reduction rate. Aebi method was used to measure CAT activity using hydrogen peroxide (30 mM) as a substratey^51^.

#### Measurement of Aβ and BDNF levels

In order to measure Aβ and BDNF levels in hippocampal tissues, the specific ELISA kits for Aβ and BDNF (ebioscience Co, San Diego, CA, USA) were used, and the instructions provided by the manufacturer were followed. The absorbances of the samples were read using a microplate reader (Biotek, USA). A standard curve was then created and the absorbance of the samples was compared to the standard curve to calculate the concentrations.

### Statistical analysis

All data are presented as means ± standard error of the mean (SEM). One-way ANOVA and Tukey's post hoc tests were used for the following factors: “Time spent in target quadrant” and all the biochemical parameters. Furthermore, Two-way ANOVA test was used for “time to find the platform” and “time to enter dark chamber”. Pearson’s correlation test was done for Vit D-biochemical parameters in UCMS groups. Differences were considered statistically significant when *P* < 0.05.
